# The Prevalence of Emphysema in Patients Undergoing Lung Cancer Screening in a Middle-Income Country

**DOI:** 10.3390/diseases13050146

**Published:** 2025-05-09

**Authors:** Marija Vukoja, Dragan Dragisic, Gordana Vujasinovic, Jelena Djekic Malbasa, Ilija Andrijevic, Goran Stojanovic, Ivan Kopitovic

**Affiliations:** 1 Institute for Pulmonary Diseases of Vojvodina, 21204 Sremska Kamenica, Serbiaivan.kopitovic@mf.uns.ac.rs (I.K.); 2 Faculty of Medicine, University of Novi Sad, 21000 Novi Sad, Serbia; 3 Faculty of Pharmacy, University Business Academy in Novi Sad, 21000 Novi Sad, Serbia

**Keywords:** emphysema, lung cancer, screening, CT, diagnosis

## Abstract

**Background:** Chronic obstructive pulmonary disease (COPD) and lung cancer are the leading causes of death globally, which share common risk factors such as age and smoking exposure. In high-income countries, low-dose computed tomography (LDCT) lung cancer screening programs have decreased lung cancer mortality and facilitated the detection of emphysema, a key radiological indicator of COPD. This study aimed to assess the prevalence of emphysema during a pilot LDCT screening program for lung cancer in a middle-income country with a high smoking prevalence. **Methods:** A secondary analysis of the Lung Cancer Screening Database of the Autonomous Province of Vojvodina, Serbia, from 20 September 2020 to 30 May 2022. Persons aged 50–74 years, with a smoking history of ≥30 pack-years/or ≥20 pack-years with additional risks (chronic lung disease, prior pneumonia, malignancy other than lung cancer, family history of lung cancer, and professional exposure to carcinogens) were offered LDCT. **Results:** Of 1288 participants, mean age of 62.1 ± 6.7 years and 535 males (41.5%), 386 (30.0%) had emphysema. The majority of patients with emphysema (301/386, 78.0%) had no prior history of chronic lung diseases. Compared to the patients without emphysema, the patients with emphysema reported more shortness of breath (140/386, 36.3% vs. 276/902, 30.6%, *p* = 0.046), chronic cough (117/386, 30.3% vs. 209/902, 23.17% *p* = 0.007), purulent sputum expectoration (70/386, 18.1% vs. 95/902, 10.53%, *p* < 0.001), and weight loss (45/386, 11.7% vs. 63/902, 7.0%, *p* = 0.005). The patients with emphysema had more exposure to smoking (pack/years, 43.8 ± 18.8 vs. 39.3 ± 18.1, *p* < 0.001) and higher prevalence of solid or semisolid lung nodules (141/386, 36.5% vs. 278/902 30.8%, *p* = 0.04). **Conclusions:** Almost one-third of the patients who underwent the LDCT screening program in a middle-income country had emphysema that was commonly undiagnosed despite being associated with a significant symptom burden. Spirometry screening should be considered in high-risk populations.

## 1. Introduction

Chronic obstructive pulmonary disease (COPD) and lung cancer are major causes of morbidity and mortality worldwide. COPD was the third and lung cancer the sixth global cause of death in 2019, with an estimated age-standardized death rate of 46.1 per 100,000 and 23.7 per 100,000 population, respectively [1]. These conditions often coexist as they share common underlying mechanisms that go beyond smoking and include gene expression and genetic susceptibility, epigenetics, chronic inflammation, and oxidative stress injury [2]. Patients with COPD have up to 6 times increased risk of lung cancer, particularly squamous cell carcinoma, and this relationship is independent of age, sex, and smoking history [3,4]. The risk of lung cancer is greatest in COPD patients who are older and have low body mass index (BMI) or emphysema [5].

Despite recent advances in oncology, the estimated 5-year survival rate for non-small cell lung cancer (NSCLC) is 26.4%, as most patients are diagnosed late [6]. Similarly, COPD symptoms are often insidious and commonly attributed to aging and deconditioning, so the majority of COPD patients remain undiagnosed and untreated until advanced stages of COPD. At this point, there is a substantial and irreversible decline in lung function, leading to a diminished quality of life and a heightened risk of severe exacerbations and mortality [7]. In recent years, lung cancer screening programs utilizing annual low-dose computed tomography (LDCT) scans for high-risk populations have facilitated the early detection of lung cancer, resulting in up to a 20% relative reduction in lung cancer-specific mortality [8]. LDCT can also be used to visualize other structural abnormalities in the lungs such as emphysema and bronchial wall thickening, which are considered a hallmark of COPD. Two separate large lung cancer screening cohorts, IELCAP (International Early Lung Cancer Program) and NLST (National Lung Screening Trial) have reported a prevalence of emphysema of 23.8% and 31%, respectively, in patients at high risk of lung cancer [9]. Importantly, most of the patients with evidence of COPD on LDCT had no prior history of chronic lung diseases.

Spirometry is the gold standard test for the diagnosis of COPD. It is a simple, non-invasive, and generally available lung function test, yet spirometric screening for COPD is not commonly performed [10]. As no large-scale spirometric screening is available, the increased use of LDCT offers new avenues for the early detection of COPD in high-risk populations. This may be of particular interest in low- and middle-income countries (LIMCs) where the smoking prevalence is much higher compared to Western countries [11]. Currently, however, lung cancer screening programs using LDCT are primarily available in selected high-income countries (HICs), and the prevalence of emphysema is not consistently reported [12,13].

Addressing the diagnosis and treatment of COPD presents a considerable and growing challenge for the health systems of developing countries. More than three-quarters of global COPD cases occur in LMICs [14]. A relative increase of 23.3% in COPD prevalence is expected from 2020 to 2050. This is largely attributed to the rising prevalence of COPD in LMICs, with an expected increase of 32.7% in LMICs compared to 3.8% in HICs [15]. This trend is likely driven by increased exposure to common COPD risk factors in LMICs, including smoking (both active and maternal), air pollution from biomass fuels, industrialization, and early childhood infections. Almost 90% of the COPD deaths in persons < 70 years of age occur in LMICs [16]. The countries with the highest COPD death rates are those in the middle range of the socio-demographic index (a composite measure of income per capita, mean years of education over the age of 15 years, and total fertility rate) [14,17]. Despite the global differences in COPD prevalence and mortality, LMICs account for only 56% of COPD’s global economic burden [18]. Additionally, 30.2% of the population in LMICs reside in countries without a national COPD guideline compared to only 1.9% in HICs. When available, the COPD guidelines in LMICs typically address active case findings less frequently than those in HICs, which likely leads to the underestimation of true COPD prevalence [19].

Given the lack of lung cancer screening programs and epidemiological data on COPD burden in developing countries, in this paper, we aimed to determine the prevalence of emphysema in high-risk populations that underwent pilot LDCT screening for lung cancer in Serbia, a middle-income country with high smoking prevalence. In addition, we aimed to compare respiratory symptom burden in high-risk populations with and without radiological evidence of emphysema.

## 2. Materials and Methods

This was a secondary analysis of the Lung Cancer Screening Database of the Secretariat for Health Care of the Autonomous Province of Vojvodina, Serbia, from 20 September 2020 to 30 May 2022. The lung cancer screening program was implemented as a pilot program in the city of Novi Sad, the capital of the Autonomous Province of Vojvodina, Serbia. According to the 2022 census, the population of the administrative area of the city of Novi Sad was 368,967 people [20]. Subjects were recruited by their primary care physicians and were offered LDCT if the following inclusion criteria were met: persons aged 50–74 years who are active smokers with a smoking history of ≥30 pack-years/or ≥20 pack-years with additional risks (chronic lung disease, prior pneumonia, malignancy other than lung cancer, family history of lung cancer and professional exposure to carcinogens); persons aged 50–74 years who stopped smoking within last 10 years from the start of screening and have the same risk as above; and no signs or symptoms of lung cancer. The exclusion criteria were as follows: CT scan within the last 12 months; previous lung cancer diagnosis; continuous use of long-term oxygen therapy; and patients with other comorbidities in the advanced stage of the primary diseases (e.g., advanced liver disease, COPD with hypoventilation and hypoxia, or congestive heart failure—New York Heart Association stage IV), where the expected length of survival due to the underlying disease limits the potential benefits of screening.

To determine the prevalence of emphysema, only a baseline LDCT scan at the time of enrollment for each subject was included in the analysis. Qualitative (visual) CT assessments were performed by a certified radiologist and documented in binary format, denoting either the presence or absence of emphysema [21,22]. The evaluation of lung nodules was performed using the Lung-RADS score (Lung Imaging Reporting and Data System) [23]. All the subjects completed a nurse-administered questionnaire containing questions on baseline demographics, smoking status, history of previous malignancies, and respiratory symptoms. Comprehensive evaluations of smoking history were documented based on the participants’ self-reports during enrollment. The smoking burden was measured using the standard metric of pack-years, calculated as the average number of packs smoked per day multiplied by the total years of smoking. In addition, we examined personal attitudes regarding tobacco smoking-related risks and willingness to stop smoking.

The study was approved by the Ethics Committee of the Institute for Pulmonary Diseases of Vojvodina, Sremska Kamenica, Serbia (No 110-V/1). Written informed consent for study participation was obtained from all the subjects involved in the study.

### Statistical Analysis

Data are expressed as the mean and standard deviations (SD) for normally distributed or as the median and interquartile range (IQR) for continuous variables with skewed distribution, and as a percentage of the total number of investigated cases for categorical variables. The patients were divided according to the presence of emphysema, and underlying characteristics were compared using a Mann–Whitney U test, Fisher exact test, Student’s *t*-test, or Chi-squared test, as appropriate. Odds ratios (ORs) and their corresponding 95% confidence intervals (CIs) were calculated to assess the strength and direction of associations between respiratory symptoms and the presence of emphysema. Univariate logistic regression analyses were performed to identify the factors significantly associated with the presence of emphysema. Variables found to be significant in the univariate analysis—namely age, gender, pack-years of smoking, and BMI—were subsequently included as covariates in the multivariate logistic regression models assessing the association between emphysema and clinical symptoms. Specifically, models were constructed to evaluate the relationship between emphysema and respiratory symptoms (shortness of breath, chronic cough, and purulent sputum expectoration), adjusting for age, gender, pack-years of smoking, and BMI. To further investigate the association between emphysema and systemic symptoms such as loss of appetite and weight loss, two separate multivariate models were developed. The first model was adjusted for age, gender, and pack-years of smoking, while the second model additionally included BMI to explore the potential mediating role of nutritional status. Odds ratios (ORs) with 95% confidence intervals (CIs) and corresponding *p*-values were reported for all the analyses. The results were considered statistically significant at *p* < 0.05.

## 3. Results

From 20 September 2020 to 30 May 2022, 1720 LDCT scans were performed in 1228 subjects. The study population included 535 males (41.5%), and the mean age for both genders was 62.1 ± 6.7 years. The prevalence of emphysema was 30.0% (386/1288). The majority of the patients with emphysema (301/386, 78.0%) had no prior history of chronic lung diseases. The differences in the baseline characteristics between the patients with and without emphysema are presented in Table 1.

**Figure 1:**
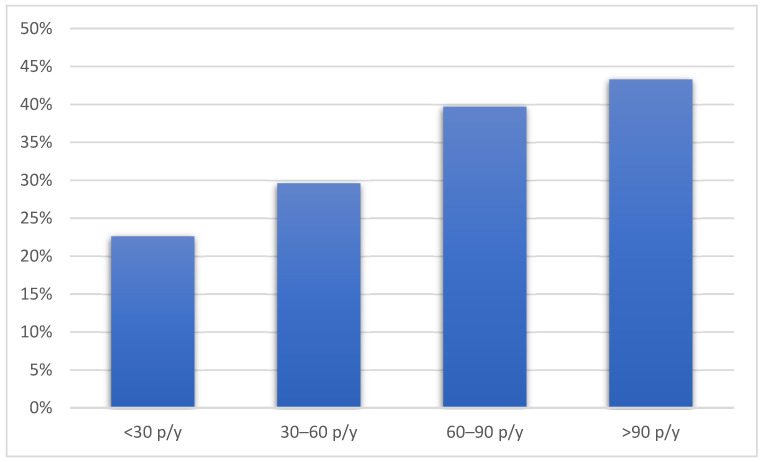
The prevalence of emphysema according to pack-years of smoking history. Figure legend: p/y—pack/years.

Compared to the patients without emphysema, the patients with emphysema were older, more frequently male, and had a lower BMI. The patients with emphysema were also more likely to be active smokers with a higher number of pack-years and used hand-rolled cigarettes more often. Pack-years of smoking were associated with the presence of emphysema in a dose-dependent manner (*p* = 0.004) (Figure 1).

**Figure 2:**
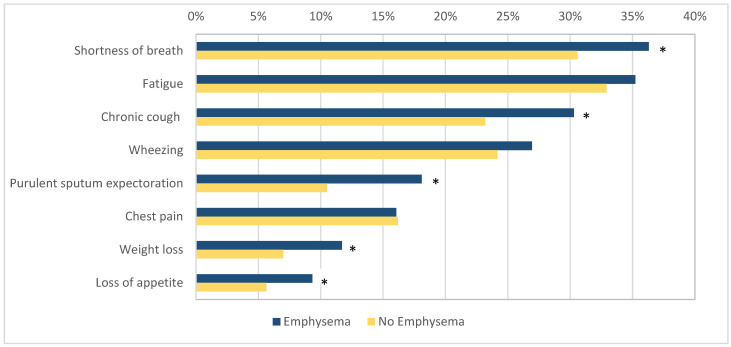
The prevalence of respiratory symptoms in patients with and without emphysema. Figure legend: *—indicates statistical significance (*p* < 0.05).

The patients with emphysema more commonly reported symptoms such as shortness of breath (140/386, 36.3% vs. 276/902, 30.6%, OR 1.29, 95% CI 1.00–1.66, *p* = 0.046), chronic cough (117/386, 30.3% vs. 209/902, 23.17%, OR 1.44, 95% CI 1.05–1.88, *p* = 0.007), purulent sputum expectoration (70/386, 18.1% vs. 95/902, 10.5%, OR 1.88, 95% CI 1.35–2.63, *p* < 0.001), loss of appetite (36/386, 9.33% vs. 51/902, 5.65%, OR 1.71, 95% CI 1.10–2.68, *p* = 0.02), and weight loss (45/386, 11.7% vs. 63/902, 7.0%, OR 1.76, 95% CI 1.17–2.62, *p* = 0.005) when compared to the patients without emphysema. No difference was found in the prevalence of wheezing (104/368, 26.9% vs. 218/902, 24.17%, OR 1.16, 95% CI 0.88–1.52, *p* = 0.29), chest pain (62/368, 16.1% vs. 146/902, 16.2%, OR 0.99, 95% CI 0.72–1.37, *p* = 0.96), and fatigue (136/368, 35.2% vs. 297/902, 32.9%, OR 1.11, 95% CI 0.86–1.42, *p* = 0.44) between the two groups (Figure 2).

In the multivariate analysis, after adjusting for age, gender, pack-years of smoking, and BMI, the presence of emphysema was significantly associated with a higher likelihood of experiencing the following respiratory symptoms: shortness of breath (adjusted odds ratio [aOR] 1.31; 95% CI: 1.01–1.71; *p* = 0.04), chronic cough (aOR 1.46; 95% CI: 1.11–1.93; *p* = 0.007), and purulent sputum expectoration (aOR 1.72; 95% CI: 1.21–2.44; *p* = 0.003). For the symptoms of loss of appetite and weight loss, two models were constructed. In the first model, adjusted for age, gender, and pack-years of smoking, emphysema was significantly associated with both loss of appetite (aOR 1.70; 95% CI: 1.08–2.67; *p* = 0.02) and weight loss (aOR 1.81; 95% CI: 1.20–2.72; *p* = 0.005). However, in a second model that additionally included BMI, these associations were attenuated and no longer statistically significant (loss of appetite: aOR 1.46; 95% CI: 0.91–2.33; *p* = 0.11; weight loss: aOR 1.32; 95% CI: 0.86–2.04; *p* = 0.19).

Almost half of the subjects in both groups were never or rarely concerned about developing lung cancer in the future (159/386, 41.2% vs. 394/902, 43.7%, *p* = 0.41, in the patients with and without emphysema, respectively). Most of the subjects were not willing to stop smoking in the near future.

The patients with emphysema had a higher prevalence of solid or semisolid lung nodules (141/386, 36.5% vs. 278/902 30.8%, *p* = 0.04) with higher RADS score (median 2 IQR 1–2 vs. median 1 IQR 0–2, *p* = 0.02). The frequency of incidental lung nodules in both groups is presented in Table 2. There was no difference in the prevalence of lung cancer between the patients with and without emphysema (8/386, 2.1% vs. 13/902, 1.4%, *p* = 0.41).

## 4. Discussion

The results of the LDCT screening program in a middle-income country demonstrated a high prevalence of emphysema in patients at risk of lung cancer. The majority of the patients with emphysema had no prior history of chronic lung diseases despite considerable symptom burden. The patients with emphysema also had a higher prevalence of incidental lung nodules as compared to patients without emphysema.

In Serbia, the use of tobacco is one of the most prevalent health risk factors. Recent estimates suggest that 36.9% of the population between the ages of 18 and 64 are daily or occasional smokers [24], a number that is significantly higher compared to the Western world [11]. The burden of smoking-related diseases such as lung cancer in Serbia is substantial and has increased over the past three decades [25]. Lung cancer is now the most common cancer among men and second most common in women with a standardized incidence rate of 68.5/100,000 and 26.7/100,000, respectively [26]. According to the 2019 Serbian National Health survey, the self-reported prevalence of COPD in Serbia is 3.5% [27]. COPD is estimated to affect 6.5% of individuals over the age of 40, totaling approximately 250,302 cases [28]. However, the true prevalence seems to be much higher. A questionnaire-based survey reported that 21.6% of the adult population in Serbia has symptoms of chronic bronchitis [29]. In a large multicenter study involving 2074 high-risk primary care patients (those with respiratory symptoms or a history of smoking), spirometry identified COPD in 22% of the participants, with half of the cases being newly diagnosed [30]. The results of our study align with these findings, showing COPD-related changes, such as emphysema on LDCT, in 30% of the cases, with nearly 80% of the patients being previously undiagnosed. Our study also revealed that the prevalence of emphysema falls within the higher range of initial reports from screening cohorts in the US, Japan, China, Israel, Italy, Spain, and Switzerland, where the prevalence of emphysema ranged from 22% to 31% [9,31,32]. However, a more recent meta-analysis reported a pooled overall prevalence of emphysema in LDCT screening cohorts at 45.3%, with a range of 19% to 78% [22]. Notably, all the studies have been conducted in high-income countries, with the exception of two studies from Brazil, a middle-income country, which report an emphysema prevalence of approximately 60% [33,34].

Globally, COPD underdiagnosis is present in 10–95% of the COPD patients. Although epidemiological data from LMICs is limited, studies from South America indicate that COPD is underdiagnosed in 71–86% of the cases [35]. Patients in LMICs face a higher risk of late COPD diagnosis, likely due to greater exposure to risk factors such as tobacco smoking and biomass fuel, as well as the underutilization of spirometry in primary care. Indeed, data from the Serbian COPD Patients’ Registry show that the majority of COPD patients are diagnosed in their 60s, with a relative value of their postbronchodilator forced expiratory volume in the first second (FEV1) of only 52.4%. In addition, these patients have a high symptom burden (COPD group B 45.3% and group D 45.6%), and almost half of them are frequent exacerbators (having ≥2 moderate or ≥1 severe acute COPD exacerbation in the previous year) [36].

Although the absolute numbers of deaths caused by COPD exceed the deaths caused by lung cancer, no systematic screening program for COPD has yet been introduced [37]. Indeed, the United States Preventive Services Task Force (USPSTF) recommends against screening for COPD in asymptomatic adults due to a lack of comparative studies on the effectiveness of screening or active case finding in COPD [10]. However, emerging evidence supports the implementation of such strategies, as a recent study by Aaron showed that the early diagnosis of COPD through active case finding with spirometry, followed by pulmonologist-directed treatment, improves lung function and quality of life while reducing subsequent health care utilization for respiratory illnesses in COPD patients [38]. In fact, the new 2024 Global Initiative for Chronic Obstructive Lung Disease Report supports active case findings in patients with symptoms or risk factors (e.g., >20 pack-years of smoking, recurrent chest infections, early life events). The report also recognizes missed opportunities to perform spirometry during lung cancer screening programs, especially when COPD-related incidental lung abnormalities are found in imaging studies [39]. We believe that the use of spirometry in high-risk cohorts could help additionally identify the patients with COPD who do not have signs of emphysema but may have different COPD phenotypes, such as chronic bronchitis. In fact, we noted a significant number of subjects who reported chronic cough and/or wheezing but lacked emphysema on LDCT.

The use of spirometry or CT scans in high-risk populations would also allow us to detect not only overt COPD, but also precursor conditions such as pre-COPD (respiratory symptoms and/or structural lung lesions without airway obstruction) or PRISm (Preserved Ratio Impaired Spirometry). The results of the EPISCAN II study from Spain showed that 22% of the population over 40 years of age have pre-COPD. These patients exhibited symptoms and CT changes comparable to those seen in COPD patients even in the absence of an obstructive spirometry pattern [40]. Two large cohorts, SPIROMICS (Subpopulations and Intermediate Outcome Measures in COPD Study) and CanCOLD (Canadian Cohort Obstructive Lung Disease), also demonstrated a significant symptom burden and limitations in daily activities among these patients [41,42]. In the Rotterdam Study, a population-based prospective cohort involving individuals aged 45 and older, the age-adjusted prevalence of PRISm was found to be 7.6% and 32.6% of those progressed to COPD during the follow-up period. Both COPD and PRISm were significant predictors of all-cause mortality, with the highest mortality observed within the first year among patients with PRISm [43]. Identifying these patients early in the course of the disease would enable timely intervention (such as smoking cessation programs) and consequently, the prevention of lung function decline [39]. This is particularly important considering that the majority of the patients in our study seem unaware of the risks associated with smoking and are generally not inclined to quit in the near future. This aligns with other studies showing that patients often downplay the health risks of smoking. Additionally, smokers with COPD appear to have less awareness of the link between their health and smoking compared to smokers without COPD [44]. In contrast, smoking cessation is shown to be the most effective and least expensive intervention for preventing COPD development and progression. It also reduces morbidity and improves survival in COPD patients [45]. Despite these facts, an estimated smoking prevalence in COPD is 38–77%, and a significant proportion of individuals with severe COPD do not quit smoking, even when facing the serious consequences of the disease [44]. While physicians tend to regularly evaluate patients’ smoking behaviors and their readiness to quit, they often lack experience in providing assistance for smoking cessation [46]. Therefore, an effective support program, whether individual or group-based, combined with a first-line pharmacological smoking cessation aid, is essential for both patients with pre-COPD, PRISm, or overt COPD. Such programs should be prioritized, especially for high-risk populations, through joint efforts by both national respiratory societies and patient advocacy groups.

Ultimately, given the complex interplay between emphysema and lung cancer, both COPD prevention and treatment may potentially affect lung cancer survival rates. Severe COPD is one of the major risk factors that precludes curative-intent surgical resection of lung cancer due to an unacceptably high risk of postoperative complications and mortality [47]. Even patients with mild to moderate COPD who undergo lung cancer surgery face a higher risk of postoperative complications compared to those with normal respiratory function [48]. In patients with stage IA lung cancer, who underwent a curative resection (lobectomy with systematic lymph node dissection), COPD was associated with a higher incidence of tumor recurrence and worse survival [49]. The presence of emphysema detected through lung cancer screening is also associated with increased lung cancer mortality at 6-year follow-up [50].

Our study has several limitations. First, we performed a qualitative assessment and did not report the extent of emphysema using quantitative CT imaging. The comparison of qualitative and quantitative findings of emphysema has been explored in a study by Amaza, which demonstrated a slight-to-fair agreement between the visual and quantitative CT assessments of emphysema [21]. However, most studies investigating the presence of emphysema in LDCT screening cohorts have relied on visual assessment, which makes our study more generalizable [22]. Also, our study was conducted in real-world settings where quantitative assessments may be unavailable, particularly in LMICs. Second, other COPD features such as bronchial wall thickening were not collected during screening and thus were not reported. Third, although emphysema is a radiological hallmark of COPD, we did not perform spirometry to confirm the diagnosis. Fourth, the study may lack sufficient power to detect certain potential associations, such as the link between emphysema and lung cancer. Fifth, high symptom burden may also be linked to the elevated prevalence of cardiovascular comorbidities in smokers, a factor that we did not account for in our study. Finally, this pilot screening program was conducted in the Province of Vojvodina and the results may not be generalizable to the whole country. Nevertheless, to our knowledge, this is the first study to report the prevalence of emphysema in a lung cancer screening program in a middle-income country in Europe, making it the second study of its kind globally, after Brazil. Understanding the global impact of COPD is important as almost 90% of the COPD deaths occur in LMICs [16]. Moreover, our study is unique in that it reports a broad range of respiratory symptoms not covered in other studies and offers valuable insights into patients’ perspectives, emphasizing the lack of awareness regarding tobacco-related health care risks.

## 5. Conclusions

In conclusion, almost one-third of patients referred to LDCT lung cancer screening programs in middle-income countries have CT evidence of emphysema. The majority of these patients had no previous history of chronic lung disease despite significant symptoms. Active case findings with spirometry should be considered in high-risk populations and patients with evidence of structural changes in LDCT images during lung cancer screening programs. This may be especially important in developing countries with a high prevalence of smoking.

## Figures and Tables

**Table 1:** The differences in the baseline characteristics between the patients with and without emphysema.

	Emphysema *N* = 386	No Emphysema *N* = 902	*p*
Age, years, mean (±SD)	63.2 (6.5)	61.6 (6.7)	<0.001
Male gender, *n* (%)	192 (49.7)	343 (38.0)	<0.001
BMI, kg/m^2^, mean (±SD)	25.8 (6.8)	28.3 (8.6)	<0.001
Education level, *n* (%)			0.8
Completed Primary Education	36 (9.3)	91 (10.1)	
Completed Secondary Education	262 (67.9)	603 (66.8)	
Completed Higher Education	82 (21.2)	199 (22.1)	
Other	6 (1.5)	9 (1)	
Active smokers, *n* (%)	340 (88.1%)	741 (82.1)	0.008
Duration of smoking, years, mean (±SD)	39.1 (8.2)	36.0 (8.7)	<0.001
Pack/year smoking, mean (±SD)	43.8 (18.8)	39.3 (18.1)	<0.001
Types of cigarettes used, *n* (%)			<0.001
Manufactured cigarettes	290 (75.3)	761 (84.6)	
Hand-rolled cigarettes	93 (24.1)	130 (14.5)	
E-cigarettes	2 (0.5)	7 (0.8)	
Pipes	0 (0)	1 (0.1)	
Exposure to second-hand smoking during childhood and adolescence, *n* (%)			0.39
Often	108 (28.0)	249 (27.6)	
Sometimes	72 (18.6)	190 (21.1)	
Rarely	59 (15.3)	109 (12.1)	
Never	147 (38.1)	354 (70.7)	
Exposure to second-hand smoking during adulthood, *n* (%)			0.25
Often	194 (50.3)	434 (48.1)	
Sometimes	87 (22.5)	235 (26.0)	
Rarely	47 (12.2)	84 (9.3)	
Never	58 (15.0)	149 (16.5)	
Willingness to quit smoking in the next 30 days, *n* (%)	90 (25.5)	178 (22.9)	0.35
Previous history of chronic lung diseases, *n* (%)	85 (22.0)	127 (14.8)	<0.001
Previous history of malignancies, *n* (%)	19 (4.29)	38 (4.21)	0.57

Abbreviations: BMI—body mass index; SD—standard deviation.

**Table 2:** The frequency of incidental lung cancer and lung nodules in the patients with and without emphysema.

	Emphysema *N* = 386	No Emphysema *N* = 902	*p*
Presence of nodules, *n* (%)	275 (71.2)	556 (61.6)	0.001
Nodule type, *n* (%)			0.008
Solid	123 (31.9)	245 (27.2)	
Semi-solid	18 (4.7)	33 (3.7)	
Ground glass	53 (13.7)	133 (14.7)	
Calcified	111 (28.8)	346 (38.4)	
RADS score, median (IQR)	2 (1–2)	1 (1–2)	0.006
Lung cancer, *n* (%)	8 (2.1)	13 (1.4)	0.41

Abbreviations: RADS—Lung Imaging Reporting and Data System; IQR—interquartile range.

## Data Availability

The datasets presented in this article are not readily available because they are part of an ongoing project and subject to privacy restrictions. Requests to access the datasets should be directed to the Secretariat for Health Care of Vojvodina.
